# Structure-Based Study to Overcome Cross-Reactivity of Novel Androgen Receptor Inhibitors

**DOI:** 10.3390/cells11182785

**Published:** 2022-09-07

**Authors:** Mariia Radaeva, Huifang Li, Eric LeBlanc, Kush Dalal, Fuqiang Ban, Fabrice Ciesielski, Bonny Chow, Helene Morin, Shannon Awrey, Kriti Singh, Paul S. Rennie, Nada Lallous, Artem Cherkasov

**Affiliations:** 1 Vancouver Prostate Centre, University of British Columbia, 2660 Oak Street, Vancouver, BC V6H 3Z6, Canada; 2 NovAliX, BioParc, 850 bld Sébastien Brant, F-67405 Illkirch, France

**Keywords:** androgen receptor, ligand binding domain, agonism, drug design, X-ray crystallography, inhibitors

## Abstract

The mutation-driven transformation of clinical anti-androgen drugs into agonists of the human androgen receptor (AR) represents a major challenge for the treatment of prostate cancer patients. To address this challenge, we have developed a novel class of inhibitors targeting the DNA-binding domain (DBD) of the receptor, which is distanced from the androgen binding site (ABS) targeted by all conventional anti-AR drugs and prone to resistant mutations. While many members of the developed 4-(4-phenylthiazol-2-yl)morpholine series of AR-DBD inhibitors demonstrated the effective suppression of wild-type AR, a few represented by 4-(4-(3-fluoro-2-methoxyphenyl)thiazol-2-yl)morpholine (VPC14368) exhibited a partial agonistic effect toward the mutated T878A form of the receptor, implying their cross-interaction with the AR ABS. To study the molecular basis of the observed cross-reactivity, we co-crystallized the T878A mutated form of the AR ligand binding domain (LBD) with a bound VPC14368 molecule. Computational modelling revealed that helix 12 of AR undergoes a characteristic shift upon VPC14368 binding causing the agonistic behaviour. Based on the obtained structural data we then designed derivatives of VPC14368 to successfully eliminate the cross-reactivity towards the AR ABS, while maintaining significant anti-AR DBD potency.

## 1. Introduction

The androgen receptor (AR) is a ligand-inducible transcription factor belonging to the nuclear receptor (NR) superfamily. The AR has a modular structure composed of an N-terminal domain (NTD), a highly conserved DNA-binding domain (DBD) and a C-terminal ligand-binding domain (LBD) [1]. Endogenous androgens—testosterone and 5α-dihydrotestosterone (DHT)—activate the receptor by binding to its androgen binding site (ABS) located in the LBD. The hormone binding initiates AR translocation into the nucleus followed by binding of the transcription factor to androgen response elements (AREs) and regulation of AR target genes, which play a crucial role in the growth, recurrence and metastasis of prostate cancer (PCa) [2,3,4].

As proliferation and survival of PCa cells are critically dependent on androgen stimulation, the AR signaling has been extensively studied as a hallmark for therapeutic targeting of PCa. This treatment usually involves some form of chemical castration to inhibit androgen synthesis (e.g., abiraterone acetate), or to prevent AR transcriptional activity (e.g., AR antagonists) [5,6,7,8,9,10]. All current clinically used AR inhibitors, including enzalutamide (Xtandi), flutamide (Eulexin), bicalutamide (Casodex) and darolutamide (Nubeqa) target the ABS of the receptor [11,12,13,14,15]. These drugs compete with endogenous androgens to bind to the receptor and block its transcriptional activity [16,17].

Abundant crystal structures of the wild-type AR-LBD in complex with agonist ligands are available in the Protein Data Bank (PDB) [18,19,20,21,22]. In contrast, to date, no crystal structure of anti-androgens bound to the AR ABS has been published. However, various studies employed computational modelling to elucidate the exact mechanism of action of antagonists [23]. Of note, mutations in the ABS are able to induce the conversion of anti-androgens from antagonists to agonists, as evidenced by crystal structures of anti-androgens with AR LBD harboring mutations such as T878A or W742L/C where the anti-androgens behave as receptor agonists [14,24]. Such gain-of-function mutations in the AR are among the main reasons for therapy-resistance in PCa [25,26]. Since therapy-resistant PCa tumours are linked to aggressive forms of the disease with high mortality rates, it is critical to understand the molecular mechanisms of AR activation through antagonistic to agonistic switch [27,28]. Several computational modelling studies (based on molecular dynamics, replica exchange and other simulations) revealed a molecular mechanism where helix 12, the most flexible part of the LBD, shifts toward the ligand binding cavity causing agonistic activation in mutant forms of the AR [29,30,31]. In fact, the dynamic nature of helix 12 has been reported to be responsible for receptor activation not only in AR but also in other members of the NR family, such as the retinoic receptor [32,33]. The specific protein-ligand interactions driving the agonistic helix 12 conformational change have been elucidated through computational modelling for bicalutamide, hydroxyflutamide and other AR modulators [29,30,31,34,35]. These findings were aimed at guiding the rational structure-based development and optimization of LDB-binding compounds resistant to antagonistic to agonistic switch [34].

Another way of bypassing the characteristic mutation-driven resistance of conventional anti-androgens that has been explored is targeting alternative drug target sites on the AR. [36,37,38]. The activation function-2 (AF2) and binding function-3 (BF3) sites in the LBD have been evaluated for new small-molecule AR inhibitors which affect co-regulator binding [39,40,41,42,43,44,45]. Additionally, compounds targeting the NTD or the DBD domains of AR have received attention, as they may affect constitutively active AR splice variants (ARVs), which lack the entire LBD and are involved in castrate resistant prostate cancer (CRPC) [46,47,48,49,50,51,52,53,54]. We have previously reported on the structure-based discovery of a series of potent small-molecule inhibitors (substituted 4-(4-phenylthiazol-2-yl)morpholines) selectively targeting the AR-DBD [49,50,54]. These compounds demonstrated marked anti-AR potency against both full-length and the constitutively active splice variant AR-V7, and the lead compound VPC-14449 effectively suppressed cell viability and AR transcriptional activity in various PCa cell models [49,50]. However, few members of this class of inhibitors demonstrated the undesired agonist effect toward the T878A mutated form of the AR at higher concentrations.

In order to understand and eliminate this undesired partial agonistic effect, in the current study, we investigated the molecular basis of the interaction between T878A AR-LBD and the most powerful partial agonist compound VPC14368 (shown in Table 1). A crystal structure of T878A-AR-LBD with VPC14368 bound to the ABS, was resolved at 1.2 Å resolution. Furthermore, we performed a computational analysis of the VPC14368 agonistic switch and observed the characteristic helix 12 shift. Here, we describe in detail the mechanism of protein–ligand interactions that enable this shift. Additionally, we report that based on this new crystal structure together with in silico drug design, new derivatives of VPC14368 were developed with chemical features that would reduce their interaction with the ABS pocket. No partial AR agonism was observed with these new compounds while their anti-AR properties were retained.

## 2. Materials and Methods

### 2.1. Enhanced Green Fluorescent Protein (eGFP) Cellular AR Transcription Assay

AR transcriptional activity was assayed as previously described [55]. Briefly, LNCaP human prostate cancer cells (LN-ARR2PB-eGFP), stably transfected with eGFP reporter driven by an androgen responsive probasin-derived promoter (ARR2PB), were grown in phenol red free RPMI 1640 media supplemented with 5% charcoal stripped serum (CSS). After 5 days, the cells were plated into a 96-well plate (35,000 cells/well) with 0.1 nM of the synthetic androgen R1881 and increasing concentrations (0−100 μM) of test compounds. Fluorescence (excitation 485 nm, emission 535 nm) was measured after 3 days of incubation with the compounds.

### 2.2. Prostate-Specific Antigen (PSA) Assay

The PSA assay was performed in parallel with the previous reporter eGFP assay and using the same plates. After 3 days incubation of LNCAP cells with R1881 and the test compound, 150 µL of the media was taken from each well and added to 150 µL of PBS. PSA levels were then evaluated using the Cobas e 411 analyzer instrument (Roche Diagnostics) according to the manufacturer’s instructions.

### 2.3. Luciferase Reporter Assay

Full-length human wild type AR was cloned into pcDNA3.1 expression plasmid (Invitrogen, Waltham, MA, USA) with a CMV promoter. The T878A mutant (T878A-AR) was generated with the Quikchange Mutagenesis Kit (Stratagene, San Diego, CA, USA) using hARWT plasmid as a template. Androgen-starved PC3 cells were seeded in 96-well plates (3000 cells/well) in RPMI 1640 + 5% CSS for 24 h followed by transfection with 50 ng of hARWT or hART878A along with 50 ng of ARR3tk-luciferase reporter construct [56] (100 ng total plasmid DNA) and 0.3 µL/well TransIT20/20 transfection reagent (TT2020, Mirus, Madison, Wisconsin, WI, USA) for 48 h. Cells were treated with increasing amounts of test compound (0.1% final DMSO concentration) and 0.1 nM R1881 for 24 h. Cell lysis was carried out with 60 µL 1X passive lysis buffer/well (Promega, Madison, Wisconsin, WI, USA). The luminescence from 20 µL of cell lysate and 50 µL of Luciferase assay reagent (Promega, Madison, Wisconsin, WI, USA) was recorded on a TECAN M200pro plate reader.

### 2.4. Biolayer Interferometry (BLI) Assay

The direct reversible interaction between small molecules and the AR was quantified by BLI using OctetRED (ForteBio, Fremont, CA, USA). The wild type and T878A-LDB in fusion with an N-terminal (His)_6_-avidin (GLNDIFEAQKIEWHE) tag were expressed in *E. coli* BL21-DE3 cells co-transformed with pBirACm plasmid (Avidity), which expresses a biotin ligase leading to the biotynilation of the avidin tag on the LBD. Cells were grown in LB media at 37 °C to an OD_600nm_ = 0.5 and then induced with 0.5 mM isopropyl-*β*-D-thiogalactoside. A measure of 20 µM of dihydrotestosterone (DHT) and 0.15 mM biotin were added and the cells were incubated at 16 °C for 18 to 20 h. Cells were then resuspended and lysed in buffer containing 500 mM Li_2_SO_4_, 50mM HEPES pH7.2, 10% Glycerol, 0.1% Octyl-beta-Glucoside, 20 µM of DHT, 5 mM β-mercaptoethanol, 10 mM imidazole and 0.1 mM phenylmethanesulfonyl fluoride. After sonication and centrifugation, the sample was loaded onto a Ni-NTA affinity column and subsequently eluted with 300 mM imidazole. After an overnight dialysis against 150 mM Li_2_SO_4_, 50 mM HEPES pH7.2, 10% Glycerol, 0.1% Octyl-beta-Glucoside, 20 µM of DHT and 0.2 mM tris(2-carboxyethyl)phosphine, purified proteins at 50 µg/mL were bound to the super-streptavidin sensors overnight at 4 °C. The assay was performed at 20 °C in the dialysis buffer supplemented with 5% dimethyl sulfoxide (DMSO) which was also used as our reference measurement.

### 2.5. Protein Production for X-ray Crystallography

The T878A-LBD of the human AR containing amino acid residues I6654-Q920 was cloned into the pET15b vector (Novagen) and expressed in fusion with an N-terminus (His)_6_ tag followed by a thrombin cleavage site (LVPRG). 100 μM of VPC14368 were added into the cell culture medium before induction. After resuspension, sonication and centrifugation, the sample was first applied to a Ni^2+^ loaded affinity column and subsequently cleaved with thrombin to remove the tag. The protein was further purified by cation exchange chromatography. All buffers used during the purification contained 50 μM of VPC14368 in order to stabilize the protein. The purified protein, stored in buffer containing 150 mM Li_2_SO_4_, 50 mM HEPES pH7.2, 10% Glycerol, 50 μM VPC14368, 0.1% Octyl-beta-glucoside, and 10 mM DTT, was concentrated to 5 mg/mL and used for crystallization trials.

### 2.6. Crystallization, Data Collection and Structure Determination

Diffracting crystals of T878A-LBD-VPC14368 complex were obtained at 22 °C in 0.1 M Hepes pH 7.5, 0.4–1 M Na citrate. Crystals were cryoprotected with 20% ethylene glycol and flash-frozen in liquid nitrogen. X-ray diffraction data were collected at the European Synchrotron Radiation Facility (ESRF) synchrotron (Grenoble, France) on ID23-1 beamline using a Pilatus 6M-F detector. Data processing and scaling were performed with XDS [57]. Crystallographic phases were determined by molecular replacement using PHASER [58] and the model was refined with REFMAC 5.5.0109 [59].

### 2.7. DHT Displacement Assay

Androgen displacement was assessed with the Polar Screen Androgen Receptor Competitor Green Assay Kit as per the instructions of the manufacturer.

### 2.8. Computational Modelling

To model the wild type (WT) AR structure, we computationally mutated the residue 878 from alanine to threonine using Molecular Operating Environment (MOE) software [60]. The modelled protein was then optimized and energy minimized using the structure preparation module built in MOE, Missing hydrogens and ionization states were added. The same procedure was applied to the mutant structure. To confirm that the binding pose of VPC14368 remains the same in the WT as in the mutant structure, we docked the ligand in both structures using Glide SP docking software with default parameters [61]. The docking grid was centered on the VPC14368 pose in the ligand binding site.

Molecular dynamics (MD) were performed using Desmond package from Schrodinger [61]. Both the modelled antagonist (WT) structures and crystallized agonist (mutant) structures were solvated using System Builder package from Schrodinger using simple point charge (SPC) water model [62] and neutralized by adding Na+ ions. The systems were built using OPLS3e force field [63]. For each of the structures, three independent 500ns MD simulations were performed using random sampling of the initial velocities to ensure a better coverage of the possible conformational changes. The simulations was carried out with 2-femtosecond time step using constant-pressure (NPT) ensemble at 300 K and pressure of 1.03 bar. Each simulation recorded 1000 frames that were then analyzed using Simulation Interactions Diagram module (Schrodinger, New York, NY, USA). The output MD trajectories were also clustered using Trajectory Frame Clustering tool (Schrodinger New York, NY, USA) setting the backbone atoms for root mean square deviation (RMSD) calculations. Representatives from three most populated clusters per each structure were used to calculate binding free energies.

Binding free energy calculations on the clustered MD snapshots were performed using Molecular Mechanics/Generalized Born Surface Area (MM/GBSA) method [64]. The energies were calculated in prime module using VSGB 2.0 solvation model [65]. The generated energy terms (e.g., Coulomb energy, H-bond terms, Van der Waals energies etc.) along with total binding free energies (dG) were averaged across the three MD snapshots of each mutant and WT complexes. Additionally, per residue energy contribution terms were estimated using Glide XP [66].

## 3. Results

### 3.1. Identification of Partial AR Agonist Properties of the VPC14368 Compound

**Figure 1:**
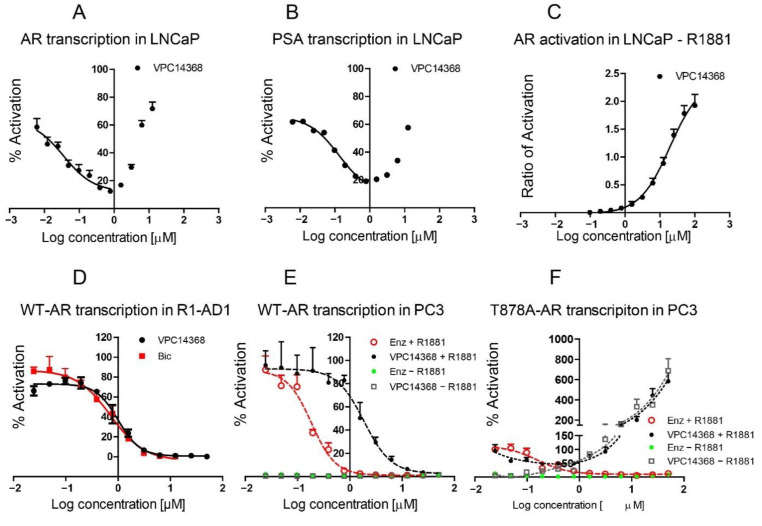
Dose–response curves (0–12.5 μM) illustrating the inhibition of the AR transcriptional activity in LNCaP cells by VPC14368 using eGFP (**A**) and PSA (**B**) assays in the presence of R1881. (**C**) Activation of AR transcription in LNCaP cells by VPC14368 in the absence of R1881. The value 1 corresponds to the eGFP signal from LNCaP cells stimulated with 0.1 nM R1881. (**D**) Inhibition of endogenous wild type AR in R1-AD1 Cells by VPC14368 and bicalutamide (Bic) in the presence of 0.1 nM R1881. Luciferase reporter assay showing AR transcriptional activity in PC3 cells transiently transfected with either WT-AR (**E**) or T878A-AR mutant (**F**). The cells were treated with either VPC14368 or enzalutamide (Enz) in the presence or absence of R1881 stimulation. Data points represent the mean of three independent experiments ± SEM. The 100% refers to luminescence recorded in 0.1% DMSO only.

To circumvent mutation-driven resistance to conventional AR inhibitors, we explored an alternative site on the DBD segment of the AR and developed a series of 4-(4-phenylthiazol-2-yl)morpholines capable of selective inhibition of the receptor′s activity by disrupting its interaction with DNA [49]. One derivative of this chemical class, compound VPC14368, demonstrated a very efficient inhibition of AR transcription in an eGFP reporter assay and of the expression of the prostate-specific antigen (PSA) in LNCaP cells, with corresponding IC50s in the nanomolar range (eGFP IC50 = 0.035 µM; PSA IC50 = 0.13 µM). However, it was also observed that this inhibition was reversed at high concentrations of the compound (Figure 1A,B), which suggested a partial agonistic effect. Indeed, in the absence of R1881 stimulation, VPC14368 enhanced the AR transcriptional activity in eGFP-expressing LNCAP cells (Figure 1C). Of note, the observed agonistic effect of this compound occurred in LNCaP cells, which carry the T878A AR mutation, and was not observed in R1-AD1 cells [67], which harbour one intact AR gene copy and express the wild-type form of the receptor (Figure 1D). In order to validate that this effect is related to the presence of the T878A mutation, a luciferase reporter assay was used to study the transcriptional activity of wild-type AR and the T878A-AR mutant in AR negative PC3 cells. AR transcription in PC3 cells transiently transfected with wild-type AR was inhibited by VPC14368 in a dose-dependent manner in the presence of R1881 and showed only a basal level of transcriptional activity in the absence of androgen (Figure 1E). In contrast, VPC14368 induced transcriptional activation of the T878A AR mutant at concentrations higher than 0.4 µM in the presence and absence of R1881 (Figure 1F). Together, these observations confirmed that VPC14368 acts as an antagonist toward wild-type AR, while it becomes an agonist in the presence of the T878A AR mutation at concentrations in the micromolar range. Interestingly, while T878A mutation alone is sufficient for agonistic switch in VPC14368, R-bicalutamide acts as an agonist only when the T878A mutation is also coupled with W742C [68].

**Figure 2:**
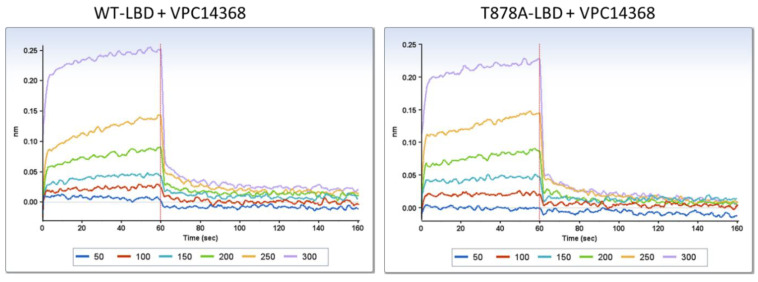
BLI dose-response curves (50–300 μM) showing the direct binding of VPC14368 to WT-LBD and T878A mutant.

It is well-documented that mutations in the ABS could render AR antagonists, such as hydroxyflutamide and R-bicalutamide, to act as AR receptor activators [14,24]. The agonistic effect of VPC14368 observed in LNCaP cells harbouring the T878A mutation suggests a similar behaviour. In order to validate if VPC14368 is able to bind to AR LBD, we tested the direct binding of this compound to a recombinant wild type LBD and a T878A mutant using Biolayer Interferometry (BLI). The binding of VPC14368 to the wild-type and mutated LBD was confirmed at high concentrations and in a concentration-dependent manner (Figure 2).

### 3.2. Structural Determination of the T878A-LBD-VPC14368 Complex

VPC14368 was able to bind the AR LBD at high concentrations. In order to elucidate the details of this interaction and eliminate this undesired agonist effect, we investigated the structural basis of the interaction between this compound and the mutated AR using X-ray crystallography. The protein corresponding to AR-LBD carrying the T878A mutation was overexpressed and purified in the presence of 100 µM and 50 µM of VPC14368, respectively. Crystals were obtained in 0.1 M Hepes pH 7.5, 0.4–1 M sodium citrate and belonged to the space group P212121. A full data set was collected at 1.2 Å resolution. Phases were obtained by molecular replacement using a wild type AR LBD structure as a search model. The asymmetric unit contains one molecule of T878A protein in complex with one molecule of VPC14368. A model was built and refined to R and R_free_ factors of 14.4 and 16.9%, respectively (Table 2).

**Figure 3:**
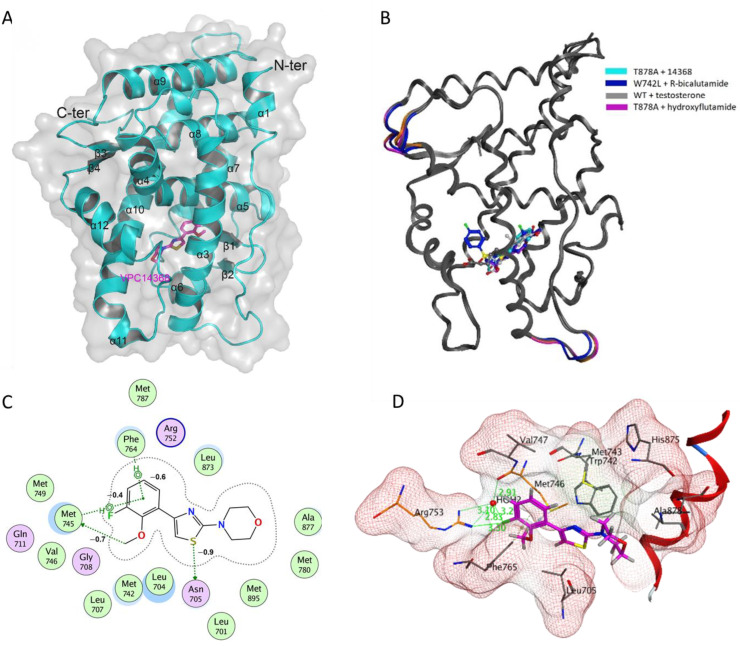
(**A**) Cartoon representation of the T878A-LBD structure in complex with VPC14368 (in magenta) shown in ball-and-stick. (**B**) Superposition of T878A-VPC14368 (in cyan) with WT-LBD-testosterone (PDB: 2AM9, in grey), T878A-hydroxyflutamide (2AX6, in magenta) and W742L-R-bicalutamide (PDB: 1Z95, in blue). Moving loops and ligands are highlighted in the corresponding colors. (**C**) Polar (pink) and hydrophobic (green) AR LBD residues interacting with VPC14368. Energies of bonds estimated with MOE are shown in kcal/mol. (**D**) Details of VPC14368 binding to the ABS. The binding is established by a few electrostatic interactions with R753 (in orange) and a water molecule (in blue) and mostly by hydrophobic interactions with surrounding residues (in grey). VPC14368 (in magenta) and protein-interacting residues are presented in ball-and-stick and the rest of the protein is shown a semi-transparent cartoon. Dashed lines indicate the hydrogen bonds and the numbers refer to distances in Å. Red ribbon represents helix 12 (residues 893–903).

According to the solved structure, the AR LBD consists of 11 α-helices arranged as a three-layered helical sandwich and four β-strands organized in two short sheets (Figure 3A)—a well-documented fold in previous structural studies of the AR-LBD. The superimposition of the solved co-crystal with the published structures of wild-type LBD-testosterone (PDB: 2AM9), T878A-hydroxyflutamide (PDB: 2AX6) and W742L-R-bicalutamide (PDB: 1Z95) (Figure 3B) complexes demonstrated their high resemblance with root mean square deviation (RMSD) values on the backbones established as 0.514 Ǻ (225 Cα), 0.195 Ǻ (229 Cα) and 0.433 Ǻ (226 Cα), respectively.

The ABS is reported to be a buried site that is mainly composed of hydrophobic side chains that interact with AR binders through Van der Waals interactions, which are essential for ligand coordination. There are also two pairs of conserved polar patches (R753 and Q712, and N706 and T878) located in opposing ends of the site, which also play a pivotal role in anchoring steroidal AR binders. In this structure (PDB ID: 8E1A), a clear electron density map allowed unambiguous fitting of the VPC14368 into the receptor′s ABS. The compound is accommodated in the ABS by a network of hydrophobic and Van der Waals interactions, as well as weak hydrogen bonds (Figure 3C,D). The phenyl ring of VPC14368 establishes hydrophobic and Van der Waals interactions with the surrounding side chains of M743, M788, M750, F765, L708, L705 and with the main chains of V747 and M746 residues. In addition, the fluorine group on the phenyl ring makes weak H-bonds with the amine of R753 and, via a water molecule with the carbonyl of M746. The thiazole ring is stabilized by hydrophobic interactions with the L705, G709 and the side chain of M746 and M743. The morpholine group interacts with the side chains of A878, F877, M743 and M781 through hydrophobic interactions and forms a weak hydrogen bond with the backbone of N706 (Figure 3C,D).

The ABS is known as an enveloped pocket with notable plasticity that can adjust the volume to accommodate ligands of various sizes. To date, there are a significant number of structures of human AR LBD solved in the presence of various steroidal and non-steroidal ligands (see structures and pdb codes in Figure S1). A few chemical classes of non-steroidal AR ligands, such as selective AR modulators, have been co-crystallized with the AR LBD, including N-Aryl-hydroxybicyclohydantoins [69], diarylhydantoins [19], and isoindolediones [70]. Otherwise, the majority of the reported AR ligands are various analogues of bicalutamide with subtle structural variations. In this regard, our VPC14368 compound represents an entirely novel chemotype in the repertoire of AR binders and can therefore provide additional insight into AR LBD binding capabilities (Figure S1). Furthermore, an analysis of protein-ligand interactions revealed that VPC14638 forms a unique set of interactions with the LBD residues when compared to the rest of the available crystal structures. Thus, VPC14368 binding mode might serve as a foundation (e.g., as a set of pharmacophore features) for structure-based screenings for novel AR modulators.

The nature of the internal flexibility of the ABS may be the cause for the promiscuity of binding of small molecules and is also likely to explain the binding of the VPC14368 to this site. In comparison with previously reported AR-ABS ligands, such as nanomolar AR inhibitors R-bicalutamide, hydroxyflutamide and bicyclic-1H-isoindole-1,3(2H)-dione, VPC14368 is a notably weaker AR binder, demonstrating binding at concentrations above 100 µM toward the target (Figure 2) [14,24,70]. The coordination of VPC14368 inside the target site is maintained by weaker electrostatic interactions with the LBD compared to those of other cited ABS antagonists. Another important observation is that highly conserved hydrogen-bond interactions with N707 and Q713 residues, which are maintained in the AR complexes with hydroxyflutamide, R-bicalutamide, and bicyclic-1H-isoindole-1,3(2H)-dione, are, in fact, missing in the AR-VPC14368 complex which likely also accounts for its much lower binding affinity.

### 3.3. Computational Analysis of the Mechanism of Agonistic Switch in the VPC14368 Mutant T878A AR LBD

To study the molecular mechanism of the agonistic switch of VPC14368, we computationally modelled the structure of the wild type (WT) structure in complex with VPC14368. First, the molecular docking of VPC14368 confirmed that the binding pose observed in the crystal structure remains largely unchanged in WT form with a slight tilting of the morpholine ring away from the native threonine 878.

**Figure 4:**
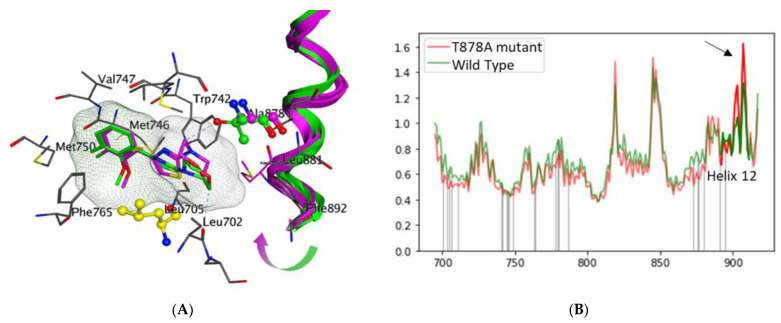
(**A**) Superimposition of helix 12 from MD clustered snapshots. Magenta–agonist; green–antagonist. The arrow shows that helix 12 shifts in the direction of the ligand binding site in the agonist state. Residue in yellow is L705. Note, VPC14368 in WT (green) is located closer to L705. A878 are also highlighted in ball and stick. (**B**) Per residue RMSD throughout the 500ns simulation averaged across 3 replicas. Residues of helix 12 are highlighted. The arrow points at the higher mobility of helix 12 in the mutant form (red).

We then performed three replicas of 500 ns molecular dynamics (MD) simulations followed by trajectory clustering of both the mutant and WT complexes. We examined the overall dynamics of the proteins and observed that while most of the residues behave similarly in WT as in mutant complexes, helix 12 is more dynamic in the mutant form (Figure 4A). This is consistent with previous findings of the importance of helix 12 for the agonistic switch [31].

To elucidate the mechanism of ligand-induced helix 12 shift, we superimposed representatives of the three most populated MD-derived clusters from each WT and T878A mutant AR-VPC14368 complexes. Importantly, all three representative WT structures had helix 12 positioned further away from the LBD site compared to all three mutant structures (Figure 4B). The morphine ring of VPC14368 was positioned closer to the L705 residue in all representatives of the WT because the native T878 forms a clash with the oxygen atom of ligand′s morphine and pushes it away. Likewise, in the mutant form, the ligand moved away from L705 residue allowing for helix 12 shift closer to the ligand-binding site (Figure 4B). An analysis of the protein–ligand contacts throughout the simulations revealed that in the mutant protein the thiazole ring forms transient interactions (maintained through 10–15% of the simulation) with W742. These interactions were not observed in any of the three replicas of WT simulations. Given that W742 is located right behind I899 belonging to helix 12, we speculate that bond formation between the ligand and W742 moves the side chain of W742 into the ligand-biding cavity allowing for subsequent dislocation of the helix 12. Therefore, an analysis of MD-derived snapshots supports the mechanism of antagonist–agonist switch through the dislocation of helix 12 presented in other studies with other AR modulators [29,34,35,71].

Interestingly, the computational analysis of R-bicalutamide agonistic switch in the presence of W742C and T878A mutations revealed that the W742 mutation reduces a clash with R-bicalutamide′s phenyl ring and enables the helix 12 shift towards the ligand binding cavity [35,71]. In contrast to R-bicalutamide, VPC14368 does not extend a functional group towards the W742 residue leaving enough space for the bulky tryptophan residue and allowing for helix 12 dislocation in the direction of ABS. This explains why VPC14368 switches to agonist in the presence of only T878A residue, while R-bicalutamide requires both W742 and T878A [68]. Additionally, in the mutant complex, the side chain of residue M896 positioned similarly to WT AR (aligned with PDB 2AMB [22]), while in R-bicalutamide-AR complex W742L mutation induces a conformational change in M896 that enables a tighter packing of helix 12. Thus, a principally different chemical scaffold of VPC14368 allows for a novel packing of AR residues that also conforms to agonistic behaviour.

Finally, we estimated the free energies of bindings and energy terms of both complexes using the MM/GBSA method. We observed that predicted ΔG of binding was comparable: −68 kcal for agonist and −71 kcal for antagonist. However, the agonist form had a slightly stronger component of hydrogen bond interactions (−0.19 vs. −0.28, agonist vs. antagonist). An increase in hydrogen bond component is likely due to transient interactions observed through MD simulations between the oxygen of ligand′s morpholine ring and T878. Of note, this observation is contrary to the Singam et al. (2019) study where they computationally analyzed various agonists and antagonists and found that agonists tend to have a stronger hydrogen bond component while antagonists tend to increase LBD stability mainly through hydrophobic interactions [29]. Thus, we further confirm that VPC14368 presents a scaffold that initiates agonistic switch in mutant form of AR through interactions different from the known compounds with dual activity.

### 3.4. Elimination of Cross-Reactivity of 4-(4-Phenylthiazol-2-yl)morpholines

Based on the structural information obtained from T878A AR LBD-VPC14368 complex, we attempted to design new derivatives with chemical features that would eliminate or reduce the binding of these compounds to the AR LBD while retaining their anti-AR activities through binding to the DBD. A number of derivatives were created to replace the benzene fragment in the studied 4-(4-phenylthiazol-2-yl)morpholines with fewer hydrophobic cycles containing various aromatic (VPC14291), aliphatic (VPC14403, VPC14406) and heterocyclic rings (VPC14449, VPC14404, VPC14435, VPC14436, VPC14439) (see Table 1).

**Figure 5:**
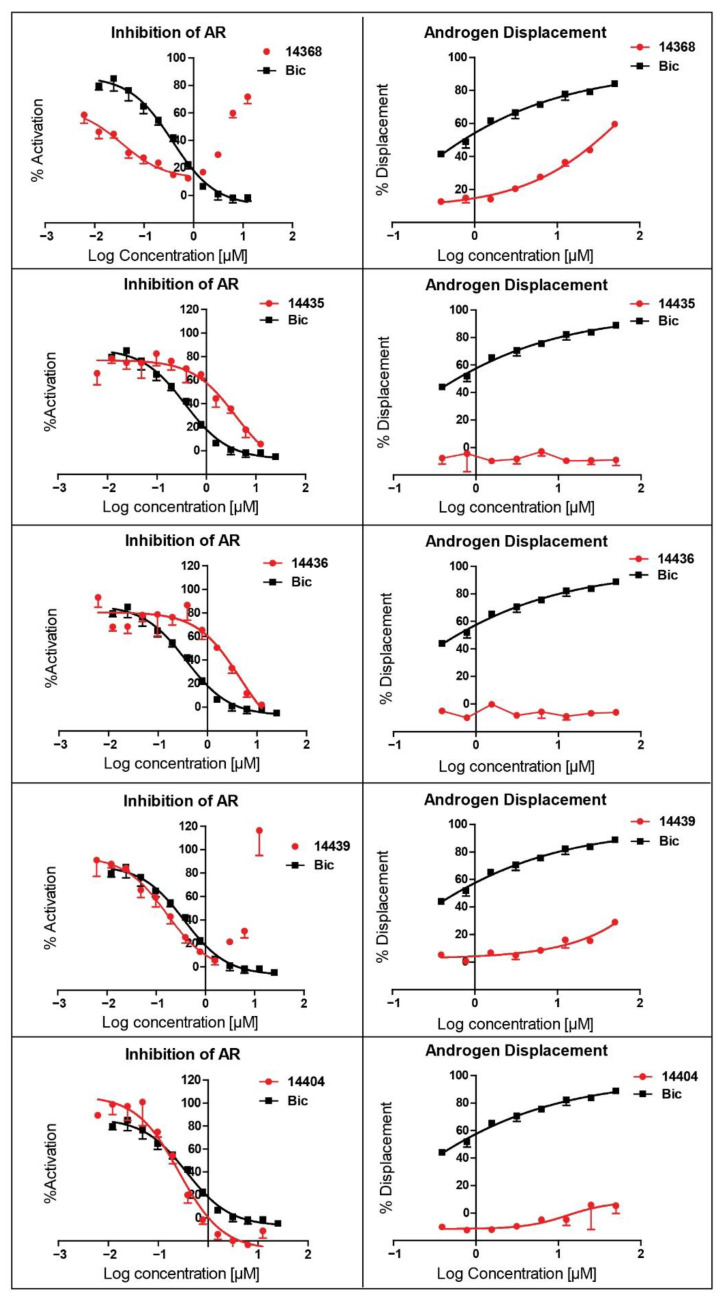
Left panel: dose-response curves of AR transcriptional inhibition by VPC14368, VPC14449, VPC14404, VPC14439, VPC14435 and bicalutamide (Bic) using eGFP AR transcriptional assay in the presence of 0.1 nM R1881. Right panel: dose-response curves of androgen displacement VPC14368, VPC14449, VPC14404, VPC14439 and VPC14435 using Polar Screen Androgen Receptor Competitor Green Assay Kit.

The ability of VPC14368 and the above derivatives to inhibit AR transcriptional activity was evaluated using the eGFP-expressing LNCAP cells. Some of the derivatives (VPC14291, 14403, and 14406) lost their anti-AR activity; however, others such as VPC14449, VPC14404 and VPC14435 maintained good potency against the mutated T878A form of AR, while no undesired partial agonistic effects were observed (Figure 5, left panel). Although VPC14435, VPC14436, and VPC14439 all possess pyridine groups with the nitrogen at the 2, 3, 4-position respectively, only VPC14439 demonstrated partial agonism at concentrations above 3 µM in the eGFP assay (Table 1 and Figure 5 left panel). The nitrogen in VPC14439 appeared to occupy a similar position to the fluorine substituent in VPC14368 and might make favourable contacts with the R753 residue, thereby contributing to the observed AR ABS interaction.

As VPC14368 was shown to bind into the ABS of LBD, this compound needs to displace the androgen prior to its LBD coordination. Therefore, we tested the ability of VPC14368 and its derivatives to displace a fluorescent androgen from the ABS using a fluorescent polarization assay (PolarScreen, Life Technologies, Carlsbad, CA, USA) (Table 1 and Figure 5 right panel). VPC14368 was indeed able to displace the androgen from the ABS with the corresponding IC_50_ established at ~30 µM. The replacement of the benzene ring with fewer hydrophobic heterocycles and bulkier aliphatic cycles could significantly reduce the androgen displacing ability of the compounds. It is noteworthy that VPC14439, which showed partial agonism in AR transcriptional assay, also demonstrated 30% androgen displacement at 50 µM (Table 1 and Figure 5).

**Figure 6:**
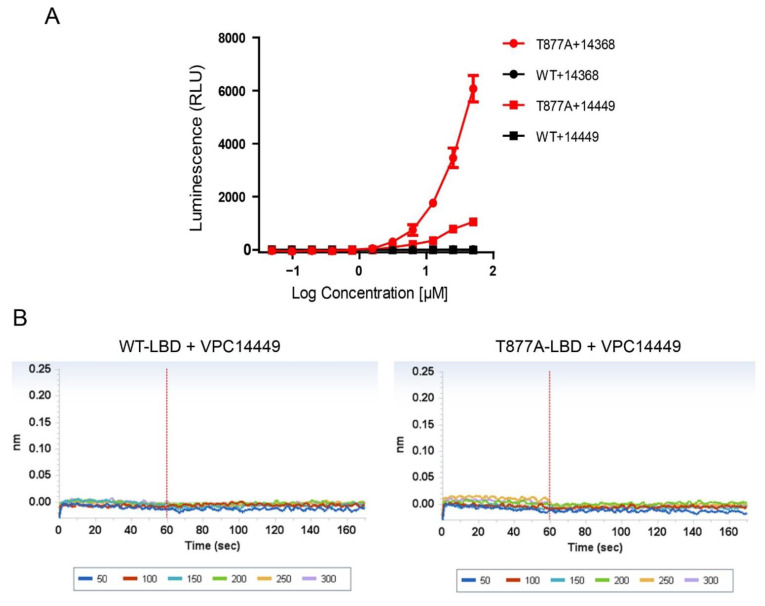
(**A**) Luciferase reporter assay showing the effect of VPC14368 and VPC14449 on AR transcriptional activity in PC3 cells transiently transfected with either WT-AR or T878A-AR mutant in the absence of R1881. (**B**) BLI curves showing that VPC14449 is not binding to wild-type-LBD neither to T878A mutant.

Among the newly designed derivatives, our previously reported VPC14449 emerged as the best candidate for selective AR DBD inhibition, demonstrating nanomolar potency range in eGFP-LNCaP cells (eGFP IC_50_ = 0.15 µM) without showing the undesired partial agonist effect toward the AR. This was also confirmed in PC3 cells transfected by the T878A-AR where the AR stimulation by VPC14449 was significantly reduced (~80% reduction) at high concentrations in the absence of R1881 in comparison with VPC14368 (Figure 6A). As well, VPC14449 did not displace DHT from the ABS (displacement < 10% at 50 µM) and in comparison to VPC14368, this compound lost the direct binding to the isolated LBD as was seen by BLI (Figure 6B).

Thus, VPC14449 was accepted as our lead DBD compound. The binding to the proposed DBD site was previously confirmed by site-directed mutagenesis experiments [49,54]. Furthermore, this compound could effectively inhibit the activity of truncated ARVs [49], which play a critical role in the development of resistance to conventional anti-androgens (the corresponding findings around VPC14449 have been discussed previously in two publications) [15,39].

## 4. Discussion

Drug resistance represents a very significant challenge to PCa drug discovery due to the emergence of gain-of-function mutations in the ABS pocket of the AR. In our previous work, we developed a new class of AR inhibitors that directly targets the DBD of the AR receptor. However, it was observed that some members of this class of AR DBD binders, such as 4-(4-(3-fluoro-2-methoxyphenyl)thiazol-2-yl)morpholine (VPC14368), demonstrated partial agonistic effect on the T878A mutated form of the AR, indicating possible cross-reactivity with the ABS pocket of the receptor. In order to eliminate this undesirable agonistic effect, VPC14368 was co-crystallized with the T878A mutated form of the AR-LBD and analyzed for structural clues to the observed partial agonism. The solved crystal structure revealed a novel chemotype of AR ABS binders that expands the repertoire of previously reported nonsteroidal AR ABS ligands (Figure S1). We also show how specific interactions with the T878A mutant LBD residues cause a conformational shift of helix 12, which has been previously linked to the agonistic switch [31,35,71]. Similarly, computational modelling of the wild type AR in complex with VPC14368 revealed the underlying molecular mechanism of the observed antagonist effect. Importantly, we elucidate the mechanism of antagonist to agonist switch of a small molecule belonging to a chemotype that has not been studied yet. This knowledge provides additional insights into molecular interactions conferring both agonist and antagonist phenotype and thus might be used to guide the structure-based design of novel small molecules targeting AR.

More importantly, the structure of the AR LBD complexed with VPC14368 provided a rational basis for developing derivatives of this chemical series such as VPC14449 that do not bind to the AR ABS and do not exhibit partial agonist effect towards the T878A mutated AR. Importantly, the designed derivatives maintain their anti-AR potency through direct DBD binding [49,54]. Thus, the results of this study provided important insights into critical structural aspects for targeting the human AR and gave us a critical rationale for improving a new class of AR inhibitors targeting the DNA-binding domain of the receptor.

## Figures and Tables

**Table 1:** Structure and activity profiles of 4-(4-(3-fluoro-2-methoxyphenyl)thiazol-2-yl)morpholine derivatives.

ID	Structure	Reporter eGFP IC_50_ (µM)	Androgen Displacement
14368	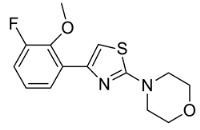	0.035 ± 0.02	Yes
14449	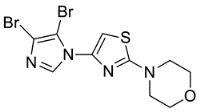	0.15 ± 0.04	<10%
14404	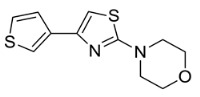	0.27 ± 0.02	<10%
14439	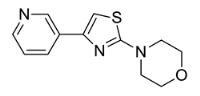	0.16 ± 0.03	Yes
14435	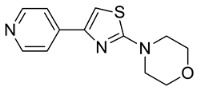	3.87 ± 1.26	No
14436	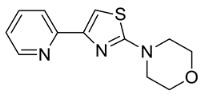	4.53 ± 0.26	No
14403	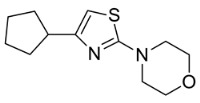	inactive	-
14406	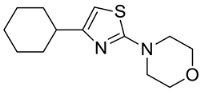	inactive	-
14291	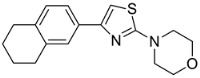	inactive	-

Reporter eGFP IC_50_-the transcriptional activity of AR measured in LNCaP cells transfected with eGFP reporter driven by an androgen-responsive probasin-derived promoter. Androgen displacement assessed with the Polar Screen Androgen Receptor Competitor Green Assay Kit.

**Table 2:** Data collection and refinement statistics.

T878A AR-LBD + VPC14368	
**PDB code**	8E1A
Expression system	*E. coli*
pH of crystallization	7.5
**Data collection**	
Wavelength (Å)	0.972452
Space group	P212121
Unit cell	a = 55.01 Å b = 65.68 Å c = 69.07 Å α = β = γ = 90°
Resolution (Å) *	40–1.2 (1.27–1.20)
Rsym (I) (%) *	4.0 (39.7)
I/σ_(I)_ *	22.84 (3.83)
Completeness (%) *	97 (86.4)
Redundancy *	6.2 (4.7)
**Refinement**	
Resolution (Å)	40–1.2
No. reflections	72,860
R_crys_/R_free_ (%)	12.4/15.1
AR molecule/AU	1
**R.m.s. deviations**	
Bond lengths (Å)	0.023
Bond angles (°)	2.15
Average B factor (Å2)	17.60
Protein (Chain A)	15.60
VPC14368 (Chain B)	13.30
Water (Chain D)	33.30
**Ramachandran plot**	
Ramachandran plot	
Favoured (%)	97.7
Allowed (%)	2.3
Outliers (%)	0

* Highest resolution shell is shown in parenthesis.

## Data Availability

We deposited the crystal structure of AR-LBD T878A in complex with VPC14368 in the Protein Data Bank under accession code 8E1A.

## Supplementary Materials

The following supporting information can be downloaded at: https://www.mdpi.com/article/10.3390/cells11182785/s1, Figure S1: Chemical structures and pdb codes of existing AR binders co-crystallized with AR-LBD.
