# Supplementary material for: Structure-Based Study to Overcome Cross-Reactivity of Novel Androgen Receptor Inhibitors
Source: Cells. 2022 Sep 7;11(18):2785. doi: 10.3390/cells11182785 (PMC9497135; doi:10.3390/cells11182785)
Supplement: Supplementary file 1 [file cells-11-02785-s001.zip › cells-1896808-supplementary.pdf]

# Supplementary Information

**Figure S1:** Chemical structures and PDB codes of existing AR binders co-crystallized with AR-LBD. The colour-coded spheres represent points of interaction with the AR LBD residues. Structural classification of co-crystallized AR binders based on the structural similarity is represented by boxes, i.e. compounds boxed together represent a group of structurally similar compounds.

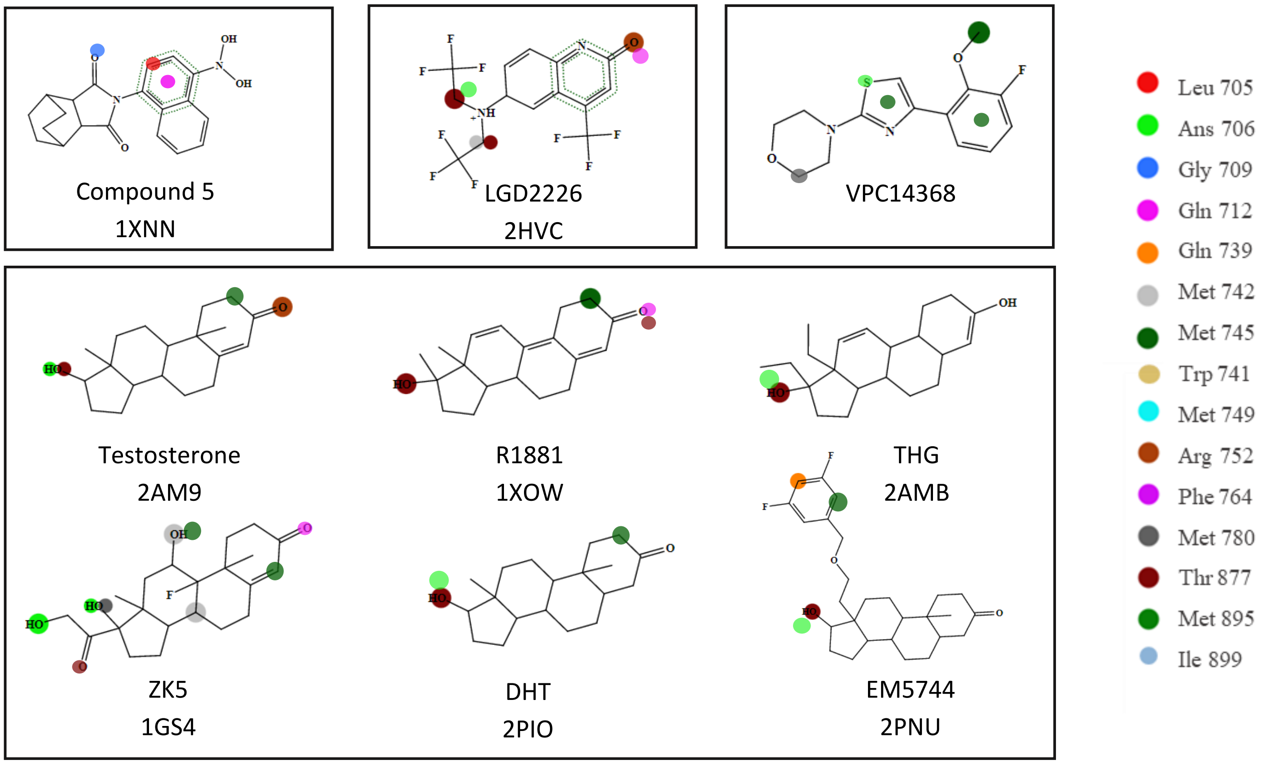

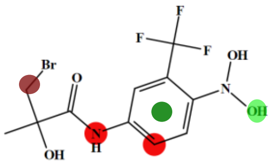

BHM
2AX9

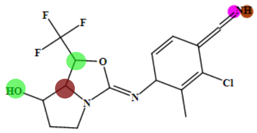

6c
3GOW

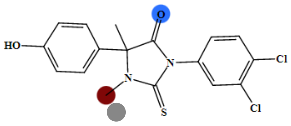

11a
3V4A

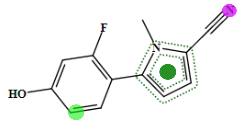

9FG
5VO4

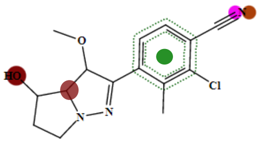

JAD
4QL8

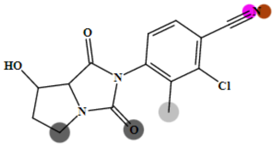

BMS-562949
2NW4

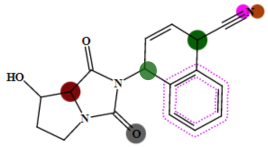

10
b
2IH
Q

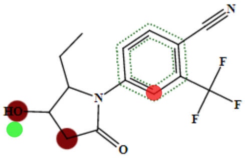

97A
5V8Q

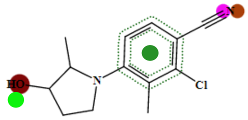

77U
5T8E

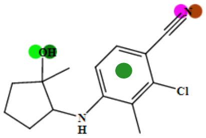

51Y
5CJ6

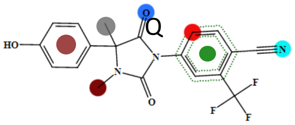

11b
3V49

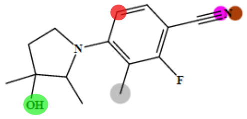

77T
5T8J

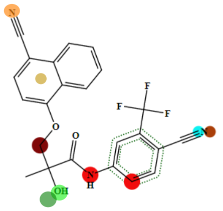

Compound-8
3RLL

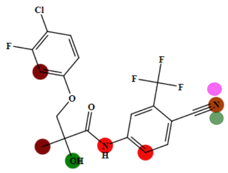

C-31
3B5R

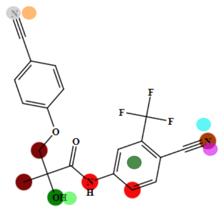

S-22
3RLJ

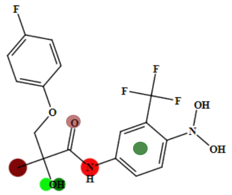

S-1
2AX7

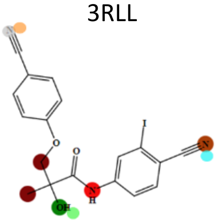

S-24
3B65

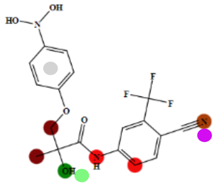

S-21
3B66

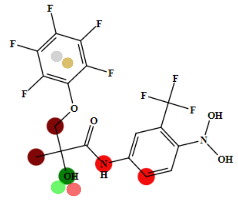

C-23
3B67

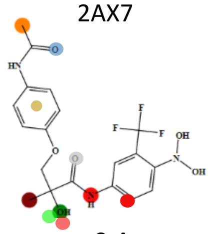

S-4
3B68

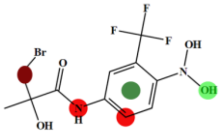

BHM (R-3)
2AX9

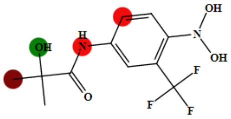

Hydroxyflutamide
2AX9

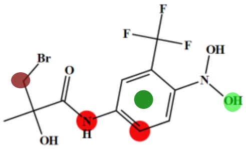

BHM
2AX9

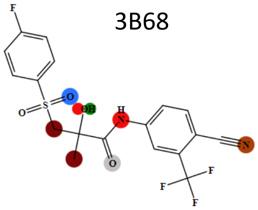

R-bicalutamide
4OKB
